# Chemical and Structural Segregation in Quaternary Ni–Cu–Fe-Co Nanoparticles: Atomistic Simulation and Experiment

**DOI:** 10.1021/acsphyschemau.5c00102

**Published:** 2025-12-08

**Authors:** Andrey Yu. Kolosov, Nikita Nepsha, Denis Sokolov, Kseniya G. Savina, Dmitry Moskovskikh, Evgenii Beletskii, Saravana Kumar M, Nickolay Yu. Sdobnyakov, Valentin Romanovski

**Affiliations:** † Department of General Physics, 64970 Tver State University, Tver 170100, Russia; ‡ Science and Research Centre of Functional Nano-Ceramics, National University of Science and Technology “MISIS”, Moscow 119049, Russia; § MllT Key Laboratory of Critical Materials Technology for New Energy Conversion and Storage, School of Chemistry and Chemical Engineering, Harbin Institute of Technology, Harbin 150001, China; ∥ Graduate Institute of Manufacturing Technology, 34877 National Taipei University of Technology, Taipei 10608, Taiwan; ⊥ Department of Mechanical Engineering, Saveetha School of Engineering, Saveetha Institute of Medical and Technical Sciences (SIMATS), Chennai, Tamil Nadu 602105, India; # Department of Materials Science and Engineering, 2358 University of Virginia, Charlottesville 22903, United States

**Keywords:** nanoparticles, quaternary nanoparticles, molecular dynamics method, Monte Carlo method, segregation, tight-binding potential

## Abstract

A comprehensive study of quaternary Ni–Cu–Fe-Co nanoparticles with sizes ranging from 2,000 to 10,000 atoms (≈10–30 nm) was carried out by combining solution combustion synthesis, X-ray diffraction (XRD), transmission electron microscopy (TEM-HAADF-EDS), and atomistic modeling (molecular dynamics and Monte Carlo simulations). Experimental XRD patterns confirmed the predominance of the face-centered cubic (*fcc*) structure with broadened reflections, indicative of nanocrystalline domains and partial coexistence of hexagonal close-packed (hcp) phases. TEM-EDS analysis showed well-defined crystallites and pronounced surface segregation of Cu (≈25–30%) enrichment relative to bulk composition and partial Co enrichment, in contrast to Ni and Fe, which concentrated in the particle cores. Molecular dynamics simulations showed that the melting temperature (*T*_m_) increases with particle size, from 1371–1379 (2000 atoms) to 1479–1488 K (10,000 atoms), corresponding to an 8.5% rise. Conversely, crystallization temperatures (Tc) decrease with faster cooling, e.g., from 1159 at 0.25 to 1086 at 0.75 K/ps, reflecting kinetic effects on solidification. The potential energy stabilized from −3.98 (2000 atoms) to −4.06 eV/atom (10,000 atoms), while surface energy decreased from 2320–2361 to 2231–2283 mJ/m^2^, in agreement with experimental evidence of Cu segregation. These combined experimental and computational insights reveal that Ni–Cu–Fe-Co nanoparticles inherently form hierarchical, labyrinth-like structures with Cu-rich shells and Ni/Fe-dominated cores.

## Graphical abstract

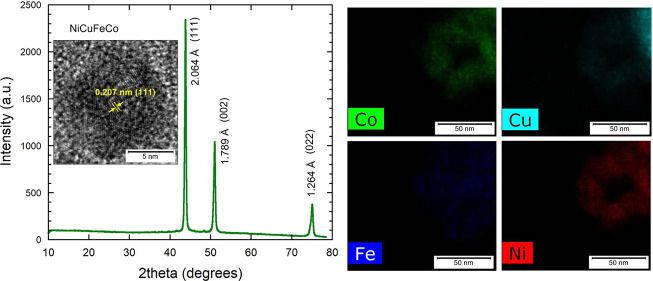

## 1 Introduction

Over the past several decades, the understanding of segregation phenomena in metallic systems has evolved significantly. Early studies on monometallic systems focused on surface and grain-boundary processes, where variations in atomic density, surface reconstruction, and defect-induced structural irregularities were first characterized. These investigations established the foundation for understanding how energetic and structural factors drive atomic redistribution at interfaces. Subsequent investigations of binary alloys revealed the thermodynamic and kinetic factors governing solute enrichment, emphasizing the influence of atomic size mismatch, cohesive energy, and surface tension on segregation behavior.^1^ Later studies on ternary systems demonstrated the emergence of more complex segregation patterns arising from the interplay of multiple atomic species, as shown in atomistic simulations of Pt–Pd–Ni nanoalloys.^2^ In recent years, attention has shifted toward multicomponent and high-entropy alloys containing four or more principal elements. In these systems, segregation occurs under the competing influences of enthalpic interactions, configurational entropy, and diffusion kinetics, resulting in nontrivial surface and interfacial structures.^3^ Modern computational approaches, including data-driven and high-dimensional modeling, have further enhanced the ability to predict segregation and disordering processes in such complex alloys.^4^ The historical transition from monometallic to binary, ternary, and now multicomponent systems reflects both the increasing complexity of alloy design and the ongoing refinement of theoretical, experimental, and computational tools for understanding segregation at the atomic scale.

In multicomponent systems such as Ni–Cu–Fe-Co nanoparticles, chemical and structural segregation is a complex problem requiring consideration of numerous factors. Chemical segregation corresponds to the nonuniform distribution of elements within a nanoparticle, where certain atoms preferentially migrate to specific regions, such as the surface, core, or grain boundaries. Structural segregation refers to the separation of regions within a nanoparticle based on different crystalline structures, such as face-centered cubic (fcc) or hexagonal close-packed (hcp).^5^ These include the influence of individual elements on the shape, crystal structure, and physicochemical characteristics of the nanoparticles. Surface composition, which can differ significantly from the internal composition, plays a significant role. These differences are due to segregation processes that determine key functional properties of the nanoparticles, including catalytic efficiency, magnetic behavior, and corrosion resistance. These characteristics, in turn, determine the wide range of applications of such nanomaterials, from catalysts to magnetic materials and superconductors.^6,7^

A key feature of multicomponent systems is their tendency toward phase instability, caused by different values of thermodynamic parameters such as cohesive energy, atomic radius, and surface energy. Rapid cooling or gas-phase condensation is often used in the synthesis of nanoparticles. This allows for the production of metastable structures with a uniform distribution of components throughout the bulk. However, the dynamics of atomic migration subsequently leads to localized elemental redistribution. Elements with lower surface energy or lower atomic density may tend to enrich the surface, thus forming a ″shell,″ while elements with higher cohesive energy tend to concentrate in the nanoparticle core.^8^

Chemical segregation in Ni–Cu–Fe–Co systems is also governed by differences in the thermodynamic properties of the constituent elements. For example, nickel and iron have relatively high cohesive energies, contributing to their stability in the bulk phase. In contrast, elements with larger atomic radii and lower cohesive energies, such as cobalt and copper, tend to migrate toward the surface.^9^ This distribution leads to the emergence of structural inhomogeneities: core–shell or gradient structures are formed, where the chemical composition varies from the center to the periphery. These changes have a significant effect on the magnetic properties of nanoparticles, since the magnetic moments of elements depend both on their local environment and on the interactions between neighboring atoms.^10^ In five-component metal nanoparticles (Ni–Cu–Fe–Co-Cr, in this case, where chromium atoms act as dopants in the nanoalloy studied), stable surface segregation of copper can also be observed.^11^ This example demonstrates that even minor compositional modifications can significantly alter segregation behavior and surface composition in related multicomponent systems.

From the perspective of structural segregation, an important aspect is the ability to form multiphase structures even with a uniform chemical composition in the bulk. Upon reaching equilibrium, atoms can reorganize to form zones with different crystal lattices or interatomic distances.^12^

In terms of practical applications, chemical and structural segregation in multicomponent nanoparticles is of great importance. In catalytic processes, altered surface composition can lead to changes in adsorption properties, thereby optimizing the reactivity of the material. For example, the presence of certain elements on the surface can promote the formation of active sites for catalyzing oxidation or reduction, as well as improve reaction selectivity.^6^ At the same time, high-magnetization materials play a crucial role in the development of spintronics, magnetic sensing, and high-density data storage technologies.^13^ Furthermore, structural segregation can promote the formation of protective oxide layers that enhance the corrosion resistance of nanoparticles, which is particularly relevant for high-temperature applications and in aggressive chemical environments.^8^

Studies of binary nanoparticles based on Ni, Cu, Fe, and Co have revealed that their magnetic and structural properties depend significantly on their composition and synthesis method. For example, Cu–Ni, Cu–Fe, and Cu–Co alloys obtained by mechanical alloying and subsequent isothermal annealing exhibit different behaviors: while the formation of a Cu–Ni solid solution leads to deterioration of magnetic properties, the precipitation of Fe and Co from supersaturated solid solutions in Cu–Fe and Cu–Co systems leads to significant improvement in magnetic characteristics, especially when Co nanoparticles are dispersed in a Cu matrix.^12^ Theoretical studies of the magnetic properties of binary alloys between Fe, Co, Ni, and Cu have shown that the formation of magnetic moments in these systems is closely related to the local chemical and magnetic environment, with transitions between high-spin and low-spin states observed in Fe-based alloys.^10^ In multicomponent systems, such as nanostructured mechanical Cu–Fe–Co alloys obtained by high-energy mechanical alloying, a nanocrystalline structure with improved magnetic properties is achieved, which expands their potential areas of application.^14^

In previous studies, we have already thoroughly studied binary nanosystems based on Ni, Cu, Fe, and Co using both experimental methods and computer modeling. In particular, in,^15–17^ the synthesis of Cu–Ni nanoparticles was carried out by exothermic combustion in solutions, and atomistic modeling of thermally induced transformations was also performed, which made it possible to identify the features of structure formation and interaction of components in nanoparticles. In another study, comparative atomistic modeling of the structure and structural transformations in Ni–Ag and Ni–Cu nanoalloys was carried out,^18^ which made it possible to establish the patterns of structural changes under various conditions. These studies provided valuable data on the mechanisms of formation and stability of binary nanoparticles, which forms the basis for further research of multicomponent systems. A comparative analysis with data on multicomponent nanoparticles, particularly those containing four elements, shows that the addition of new components significantly complicates the nature of interatomic bonds and facilitates structural rearrangements. These changes can be both beneficial - due to targeted modification of the material properties - and detrimental, leading, for example, to increased brittleness^19^ or instability upon heating.^20,21^ At the same time, effective control of structure formation processes in such systems requires strictly controlled synthesis conditions and a thorough understanding of the nature of interatomic interactions for the targeted formation of desired physicochemical properties.

Clearly, the patterns of chemical and structural segregation processes in quaternary Ni–Cu–Fe-Co systems will be significantly more complex than in the corresponding bimetallic or ternary analogs. In this work, we focus on the quaternary Ni–Cu–Fe–Co system, which serves as a representative model of multicomponent metallic nanoparticles combining elements with contrasting thermodynamic and magnetic characteristics. The interaction of four different atomic species leads to the formation of a diverse range of microstructures due to the complex interplay between their thermodynamic and structural properties. In Ni–Cu–Fe–Co systems, differences in cohesive energy, atomic size, and mixing enthalpy influence the degree of chemical ordering, surface segregation, and phase separation during solidification or thermal treatment. As a result, these multicomponent alloys can exhibit uniformly mixed solid-solution phases when the constituent elements are mutually soluble. Conversely, when there are significant disparities in atomic radius or bonding strength, the system tends to develop pronounced structural heterogeneity, such as compositional clustering, core–shell architectures, or chemically enriched domains at the surface or grain boundaries. This variability in microstructural evolution enables a broad spectrum of physical properties and functional performance. In some cases, effects similar to those characteristic of high-entropy alloys are observed.^22^ Moreover, even minor changes in the relative content of components or in synthesis conditions can lead to radically different segregation regimes, requiring an integrated approach for their study and subsequent control. As demonstrated in,^23–25^ segregation processes in multicomponent metallic systems can be interpreted through how each elemental species contributes to the formation of the final configuration during melting, crystallization, and coalescence. Atomistic simulations using molecular dynamics (MD) and Monte Carlo approaches show that certain metals preferentially occupy sites of higher coordination during solidification, thereby forming the core region of the nanoparticle and stabilizing its interior. Others exhibit a stronger affinity for under-coordinated environments and segregate toward the surface or near-surface layers, while a third group displays weak segregation driving forces, maintaining a relatively uniform spatial distribution across the nanoparticle volume. This classification is not merely conceptual: it arises from quantitative analysis of local energies, coordination statistics, and diffusion behavior under varying particle sizes and cooling rates, and it enables reliable prediction of which elements govern surface chemistry and which stabilize the crystalline framework in complex nanoalloys.^23–25^

The aim of this work is to investigate the effect of the size of four-component Ni–Cu–Fe-Co nanoparticles and the cooling rate on chemical/structural segregation processes and phase transformations using molecular dynamics.

## 2 Materials and Methods

### 2.1 Materials and Reagents

To synthesize Ni–Cu–Fe-Co high-entropy alloy nanoparticles (HEA-NPs), the next precursor mixture was used: nickel nitrate hexahydrate (Ni­(NO_3_)_2_·6H_2_O, 98%; Sigma-Aldrich, USA), cobalt nitrate hexahydrate (Co­(NO_3_)_2_·6H_2_O, 98%; Sigma-Aldrich, USA), copper nitrate trihydrate (Cu­(NO_3_)_2_·3H_2_O, 99.99%; Sigma-Aldrich, USA), and iron nitrate nonahydrate (Fe­(NO_3_)_3_·9H_2_O, 98%; Sigma-Aldrich, USA). Hexamethylenetetramine (C_6_H_12_N_4_, 98%; Sigma-Aldrich, USA) was used as a reducing agent.

### 2.2 Materials Synthesis

The synthesis of Ni–Cu–Fe-Co HEA-NP suspension was performed via a two-step procedure. Initially, a homogeneous solid solution of the nanoparticles was produced using a sol–gel combustion technique.^26,27^ Experimentally, the nanoparticles were synthesized from an equiatomic precursor mixture, ensuring an atomic ratio of Ni:Cu:Fe:Co = 1:1:1:1. Equiatomic amounts of metal nitrates were dissolved in distilled water in a ceramic vessel and thoroughly stirred to ensure uniform mixing. Subsequently, organic fuel was incrementally introduced under continuous agitation. Hexamethylenetetramine (HMTA, C_6_H_12_N_4_) served as the organic fuel. The fuel-to-oxidizer ratio, calculated according to the total oxidizing and reducing valences of the reactants, was maintained at 1.0, ensuring stoichiometric combustion and complete conversion of metal nitrates. The resulting mixture was then dried in ambient air at 80 °C for 24 h, allowing complete removal of residual moisture and formation of a gel-like intermediate.^28,29^ The resulting gel-like intermediate exhibited a homogeneous xerogel structure composed of uniformly distributed metal–organic complexes and retained residual porosity typical of sol–gel precursors. This precursor underwent combustion initiated by a resistively heated wire inside a constant-pressure reactor under an inert argon atmosphere, triggering a rapid, self-propagating exothermic reaction throughout the gel. The process yielded a finely dispersed polymetallic nanopowder.^30,31^

The combustion reaction proceeded spontaneously after ignition without external heating control; the highly exothermic front developed and quenched within 1–10 s, implying extremely high transient heating and cooling rates characteristic of solution-combustion synthesis. Direct in situ measurements of the temperature–time profile of this gel were not performed in this study. However, according to reviews on solution-combustion synthesis, the self-sustaining front in such sol–gel systems occurs very rapidly (fractions of a second to a few seconds) and causes a short-term increase in the mixture temperature to high values (reviews cite typical maxima ranging from several hundred to >1000 °C, depending on the composition and reaction conditions). Therefore, the reaction temperature and time estimates given in the text are based on literature data and not the result of direct thermometric measurements in our sample.

### 2.3 Materials Characterization

Structural and phase characterization of the synthesized nanocrystallites was carried out using X-ray diffraction (XRD) on a DIFREY-401 diffractometer operating at 25 kV and 40 mA. The instrument employed Cr–Kα radiation in Bragg–Brentano geometry, with subsequent conversion to Cu–Kα for analysis. Diffraction data were processed using Jade software, which was also used to determine lattice constants and estimate crystallite dimensions of the HEA-NPs. The diffractometer operated with a step size of 0.02° 2θ and a counting time of 1 s per step, providing a detection sensitivity of approximately 3–5 wt % for crystalline phases and allowing identification of coherent domains as small as ∼ 2 nm.

Morphological assessment of the particles was performed via transmission electron microscopy (TEM) using a JEM-2100F microscope equipped with an energy-dispersive X-ray spectroscopy (EDS) detector (EDAX Genesis XM 460). The system operated within a voltage range of 80–200 kV, offering a lateral resolution of at least 0.14 nm. Image analysis was conducted using ImageJ software.

### 2.4 Problem Statement and Modeling Methodology

This study examined Ni–Cu–Fe-Co nanoparticles with equal atomic ratios (Figure 1). Models of varying sizes containing 2,000; 5,000; and 10,000 atoms were analyzed using molecular dynamics simulation. The initial configurations obtained using the software^32^ were characterized by a uniform distribution of atoms and a predominant *fcc* structure. During the simulation, the nanoparticles were heated from 300 to 1800 K, then cooled to 300 K, after which their structure was analyzed. The following parameters were used in the molecular dynamics experiment: relaxation time was 15 ps, the rate of temperature change for heating and cooling was 0.25, 0.5, and 0.75 K/ps. The representative cooling rates, close to 1 K/ps, correspond well to experimentally achievable values in rapid-cooling or ion-assisted deposition processes,^33^ ensuring that the simulation parameters realistically reproduce the physical cooling behavior characteristic of nanomaterial synthesis.

**Figure 1:**
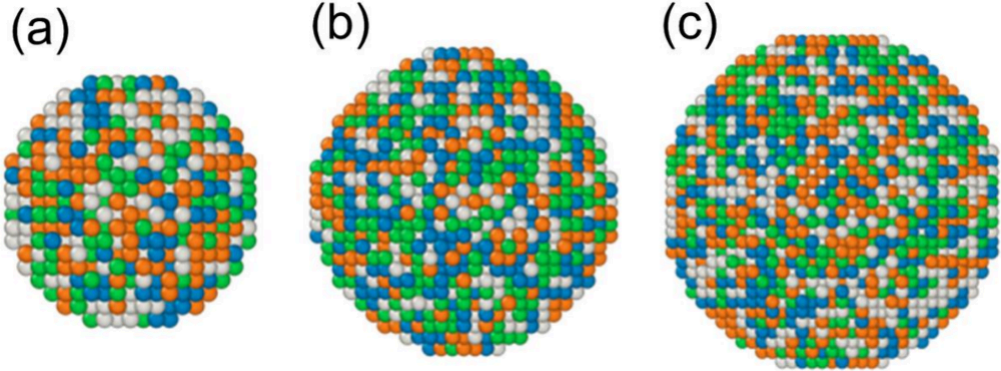
Initial configurations of equiatomic Ni–Cu–Fe–Co nanoparticles: (a) 2000 atoms, (b) 5000 atoms, and (c) 10,000 atoms. Blue atoms represent iron, brown for copper, gray for cobalt, and green for nickel.

MD simulations were performed using proprietary software that utilizes a soft stochastic thermostat,^34^ as well as LAMMPS software^35^ with a Nosé-Hoover thermostat.^36,37^ The tight-binding potential (TBP) was also used in this study.^38,39^ The Lorenz-Berthelot rule was used to obtain the TBP cross-parameters. Table 1 summarizes the main parameters used in the molecular dynamics simulations. Here r_0_ is the first-neighbor distance in the lattice, A is an effective parameter in the sum of Born-Mayer ion–ion repulsions, ζ is an effective hopping integral, and q describes its dependence on the relative interatomic distance, the parameter p, still depending on the interacting atomic species only, should be related to the compressibility of the bulk metal.^38^ The cutoff radius for the tight-binding potential in this work was 7.55 Å for all elements.

**Table 1:** Parameters for the Tight-Binding Potential^38,39^

bonds	A, eV	ζ, eV	p	q	r_0_, Å
Co–Co	0.095	1.488	11.604	2.286	2.5131
Cu–Cu	0.0855	1.224	10.96	2.278	2.556
Fe–Fe	0.1184	1.5418	10.7613	2.0379	2.4824
Ni–Ni	0.0376	1.07	16.999	1.189	2.4918
Co–Cu	0.0901	1.3496	11.282	2.282	2.5346
Co–Fe	0.1061	1.5147	11.1826	2.162	2.4978
Co–Ni	0.0598	1.2618	14.3015	1.7375	2.5025
Cu–Fe	0.1006	1.3737	10.8607	2.158	2.5192
Cu–Ni	0.0567	1.1444	13.9795	1.7335	2.5239
Fe–Ni	0.0667	1.2844	13.8802	1.6135	2.4871

In addition, the results were further verified using Monte Carlo (MC) simulations.^32^ In the framework of the computer simulation by the Monte Carlo method, 10^6^ steps were performed with a temperature step of 0.5 K. Structural analysis of nanoparticles after the completion of the crystallization process was carried out using the OVITO software package, using the method of matching with polyhedral templates.^40^

Surface energy is one of the factors ensuring the stability of nanoparticles^41^ and also determines the patterns of chemical segregation in multicomponent nanoparticles.^42^ In this study, two methods for estimating the surface area of nanoparticles were used to determine the surface energy: 1) Using the OVITO software^40^ to analyze atomistic models of nanoparticles, in particular, to determine their surfaces, for example, using the alpha-shape method^43^ with a smoothing algorithm.^44^ 2) Using Voro++ software,^45^ which performs three-dimensional tiling of space with convex polyhedra using the Voronoi method. The control parameter for extracting surface atoms is the number of atomic layers (in this work, one atomic layer).

The next task was to directly calculate the surface energy. For this, a modified formula^46^ is used, which considers the multicomponent nature of the nanoparticle:

$$\sigma_{s}=\frac{1}{A_{0}}\overset{N_{\alpha }}{\underset{i=1}{\sum }}\left(E_{i}-E_{0}\right)$$

where A_0_ is the area of the dividing surface, index α numbers the components of the nanoparticle, index i runs from 1 to N_α_ and numbers the index of the atom of the α component of the surface phase, N_α_ is the number of atoms of the α component in the surface phase,E_0_ is the average energy of the bulk phase (excluding components).

## 3 Results and Discussion

### 3.1 Atomistic Simulations of Ni–Cu–Fe–Co NP Structure Formation

Molecular dynamics simulations were performed to investigate quaternary Ni–Cu–Fe–Co nanoparticles of various sizes, ranging from 2000 to 10,000 atoms, at different temperature change rates of 0.25, 0.5, and 0.75 K/ps. The simulation results were subsequently processed and visualized using the software packages described previously.^40–45^ The temperature dependence of the potential component of the specific internal energy was determined to analyze phase transitions and assess thermal stability. Furthermore, the radial distribution of local atomic densities for each component was evaluated as a function of the radius of gyration to examine elemental segregation. A comprehensive analysis of the final nanoparticle structures, including surface energy calculations, was conducted to provide deeper insight into the structural and energetic characteristics of the synthesized nanoparticles.

Figures 2–4 (a, c, e) show the caloric curves for the corresponding sizes, and Figures 2–4 (b, d, f) show the local densities of the final nanoparticles.

**Figure 2:**
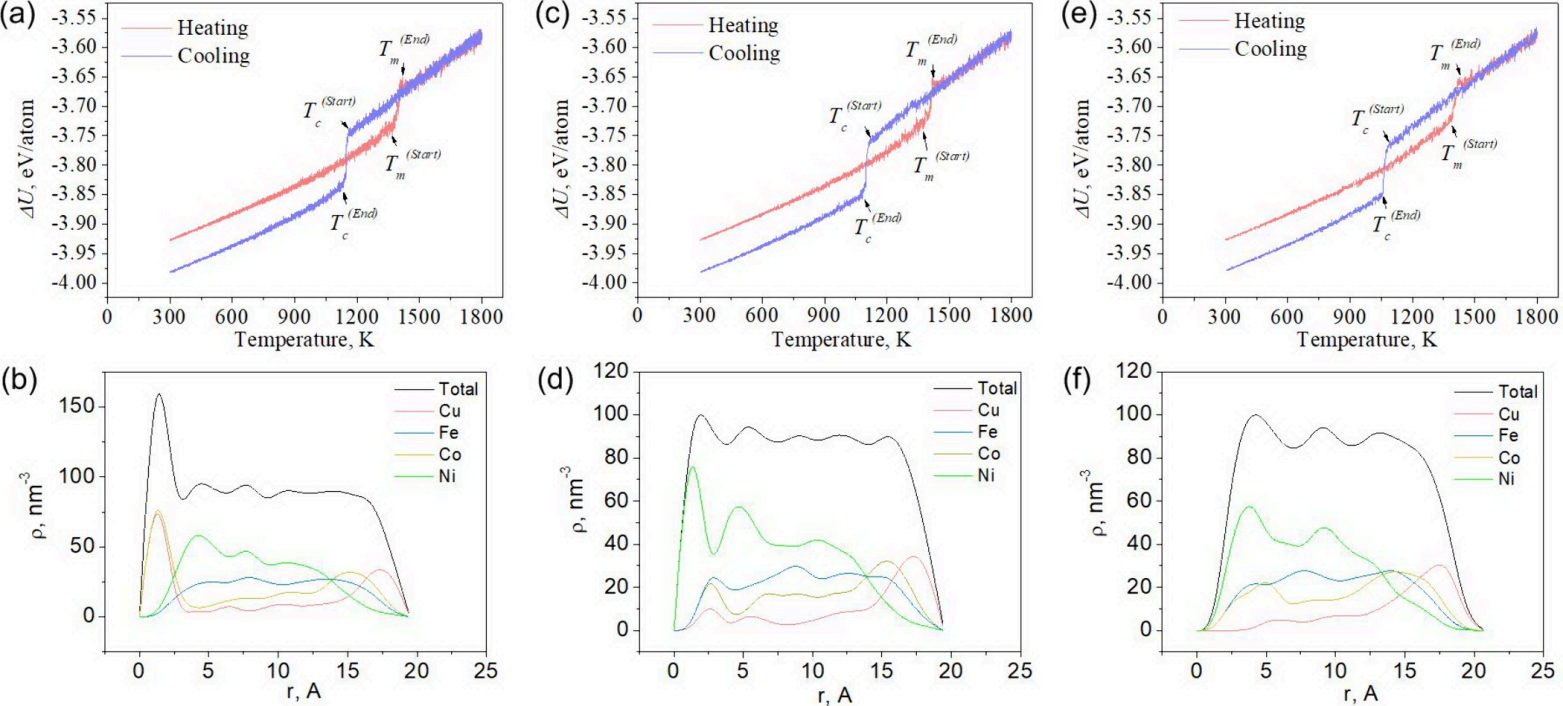
Dependence of the potential part of the specific internal energy on temperature (a, c, e) and the dependence of the local density on the radius of gyration (b, d, f) at different rates of temperature change for the Ni–Cu–Fe–Co nanosystem containing 2000 atoms. (a, b) 0.25, (c, d) 0.5, and (e, f) 0.75 K/ps.

**3:**
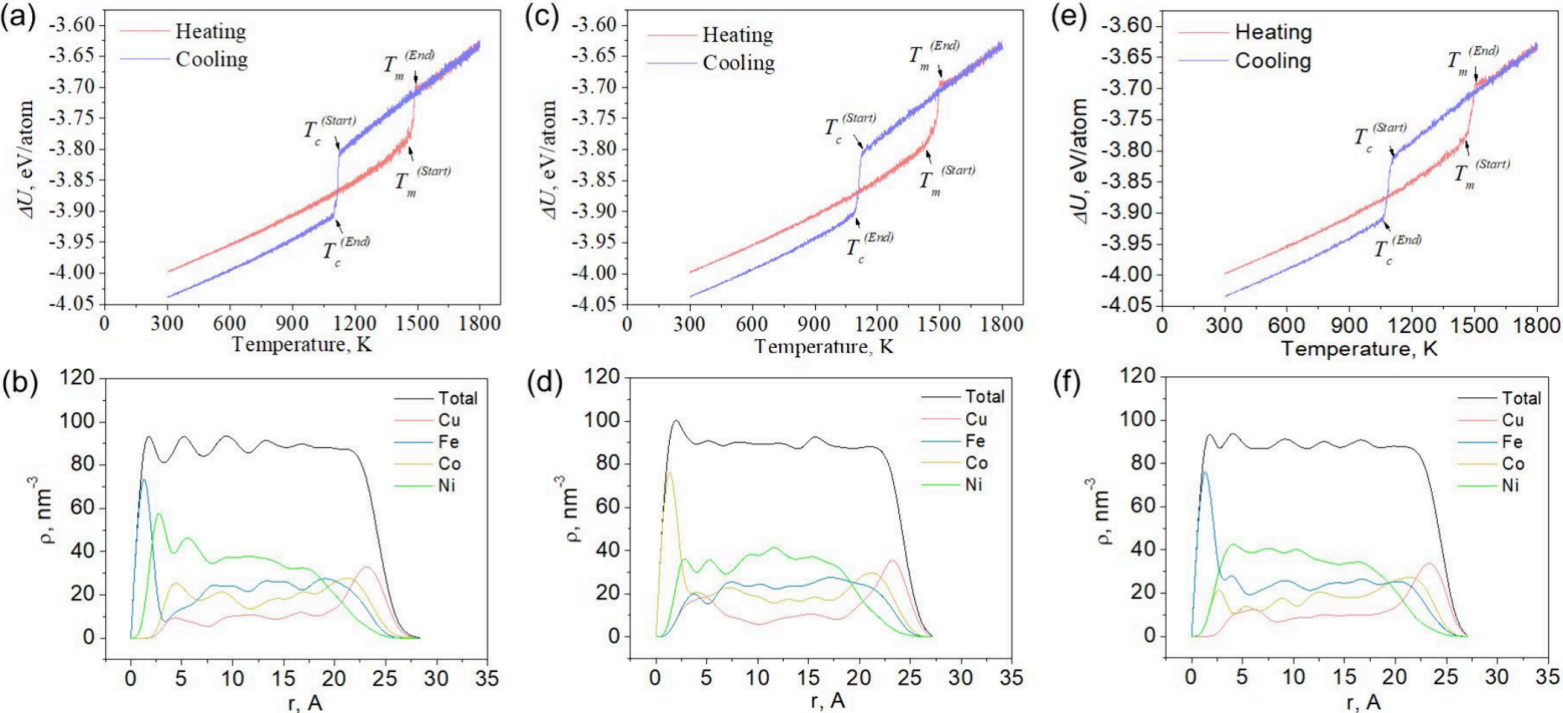
Dependence of the potential part of the specific internal energy on temperature (a, c, e) and the dependence of the local density on the radius of gyration (b, d, f) at different rates of temperature change for the Ni–Cu–Fe–Co nanosystem containing 5,000 atoms. (a, b) 0.25, (c, d) 0.5, and (e, f) 0.75 K/ps.

**Figure 4:**
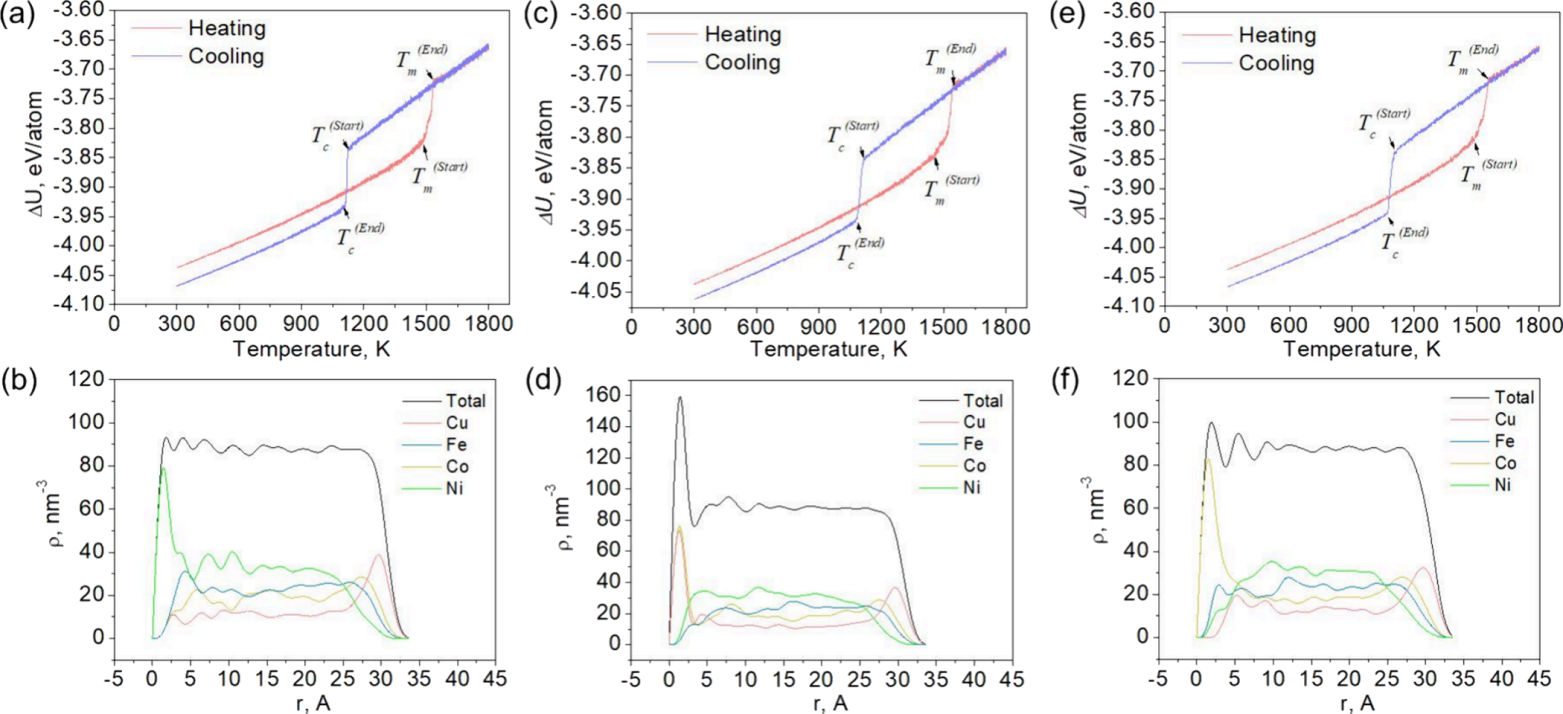
Dependence of the potential part of the specific internal energy on temperature (a, c, e) and the dependence of the local density on the radius of gyration (b, d, f) at different rates of temperature change for the Ni–Cu–Fe–Co nanosystem containing 10,000 atoms. (a, b) 0.25, (c, d) 0.5, and (e, f) 0.75 K/ps.

A study of cooled Ni–Cu–Fe-Co molten nanoparticle of various equiatomic compositions revealed patterns in phase transitions and energy characteristics. The study identified key characteristics, including melting and crystallization temperatures, the potential energy of the final configurations, the surface energy of the nanoparticles, and the dependence of local density on the radius of gyration. Analysis of the results revealed that the melting temperature (Tm) rises with increasing nanoparticle size: for systems of 2,000 atoms, it is 1371–1379 K, for 5,000 atoms, it is 1448–1453 K, and for 10,000 atoms, it reaches 1479–1488 K, representing an increase of 8.53%. Conversely, the crystallization temperature (Tc) drops with increasing cooling rate: for nanoparticles of 2,000 atoms, Tc decreases from 1159 at 0.25 K/ps to 1086 at 0.75 K/ps, which corresponds to a decrease of 6.3%, indicating a pronounced influence of kinetic factors on the crystallization process.

During cooling, the nanoparticles exhibit atomic segregation: a copper- and cobalt-rich layer with a lower iron content forms on the surface, while the inner layers are predominantly composed of nickel, iron, and cobalt. Co is present both in the shell (along with Cu) and in the core (along with Fe and Ni), although its segregation to the surface is less pronounced than that of Cu. This atomic distribution is illustrated by Figures 2–4 (b, d, f), which show the dependence of the local density of individual atomic species on the radius of gyration, and Figure 5, which depicts a Ni–Cu–Fe–Co nanoparticle containing 10,000 atoms.

**Figure 5:**
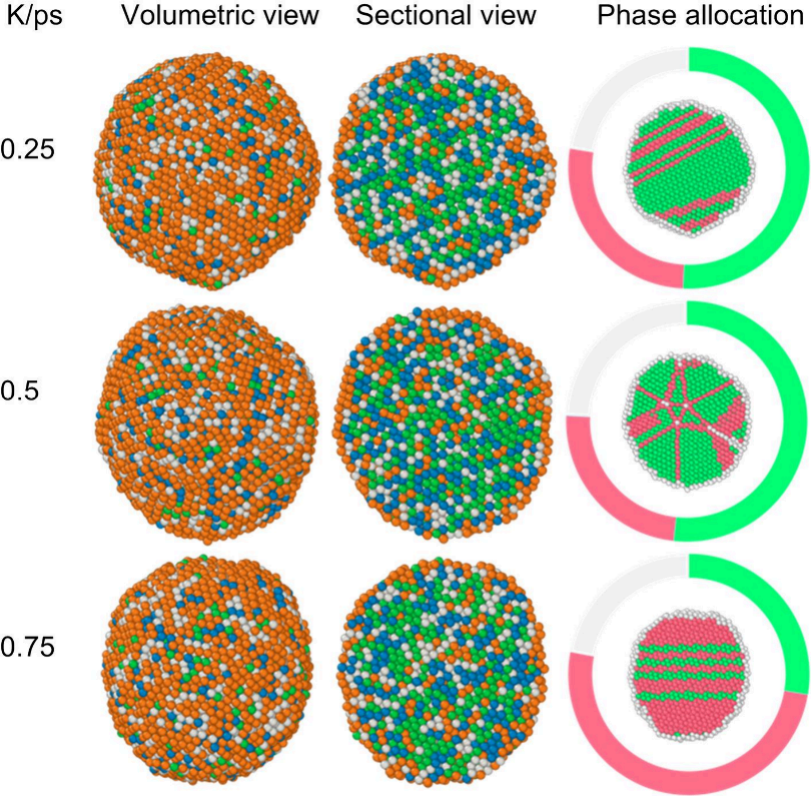
Instantaneous configurations of the Ni–Cu–Fe–Co nanosystem containing 10,000 atoms at a final temperature of 300 K. Shown are volume view, sectional view (designations correspond to Figure 1), and the phase distribution, where green atoms are fcc lattice, red ones are hcp, and white ones are unrecognized.

The potential component of the specific internal energy (ΔU) shows a decreasing trend with increasing particle size: for 2,000 atoms, it is −3.98 eV/atom, for 5,000 atoms – −4.03 eV/atom, and for 10,000 atoms – −4.06 eV/atom, indicating structural stabilization with increasing nanoparticle size (see Figure 2–4 a, c, e). Similarly, the surface energy (σs) decreases: from 2320 to 2361 mJ/m^2^ for 2,000 atoms to 2231–2283 mJ/m^2^ for 10,000 atoms (see Table 2). However, surface energy values are largely determined by the choice of the dividing surface in the nanoparticle, which can lead to either an increase or decrease in surface energy with increasing size.^47–50^

**Table 2:** Melting and Crystallization Onset Temperatures, as well as the Potential Portion of the Specific Internal Energy and Surface Energy of the Final Configurations of Ni–Cu–Fe-Co Nanosystems at Different Rates of Temperature Change (the Error in the Values of Surface Energy from a Series of MD Experiments Is No More Than 2%)

2000 atoms					
speed, K/ps	*T*_m_, K	*T*_c_, K	Δ*T* = *T*_m_ – *T*_c_, K	Δ*U*, eV/atom	σ_s_, mJ/m^2^
0.25	1371	1159	212	–3.98152	2320
0.5	1374	1118	256	–3.98298	2361
0.75	1379	1086	293	–3.97873	2349

5000 atoms					
0.25	1448	1135	313	–4.03907	2319
0.5	1450	1130	320	–4.03625	2322
0.75	1453	1112	341	–4.03331	2274

10,000 atoms					
0.25	1479	1126	353	–4.06845	2283
0.5	1481	1119	362	–4.06167	2231
0.75	1488	1107	381	–4.06659	2250

To compare our surface energy data with experimental data (Table 3), we will use,^51^ as it provides surface energy values for a number of metals in the solid and liquid states, as well as the corresponding temperature derivatives, with the exception of the temperature derivative for γ-Fe. The values recommended in^51^ are generally in good agreement with existing estimates by other authors, including experimental ones.^52–58^ So, the data presented in Table 3 are intended to illustrate the principal differences in surface energies based on the available experimental data.

**Table 3:** Estimated Macroscopic Surface Energies of Elements at *T* = 300 K^a^

element	*T*_*m*_, K [15]	σ_*s*_ (*T*_*m*_), mJ/m^2^	dσ_*s*_/d*T*, mJ/(m^2·^K)	σ_*s*_ (*T*), mJ/m^2^
Cu	1358	1473	–0.50	2002
Ni	1728	1920	–0.50	2634
β-Co	1768	2404	–0.17	2654
γ-Fe	1811	2170	–0.21*/ −0.52**	2487*/2956**

a *Considering the range of values given in^59^ (0.23–0.565 mJ/(m^2^·K)), for temperatures *T* < *T*_m_ as the upper limit, based on available data^51^ that the values dσ_l_/dT for γ-Fe and δ-Fe are equal, and also considering estimates σ_s_ at 0 K^60^ at the level of 2550 mJ/m^2^. **Considering DFT data 3120 mJ/m^2^.

Thus, the estimated surface energies of the Ni–Cu–Fe-Co nanoalloy components make it possible to assess both the size effect for the studied configuration and the influence of segregation processes on surface energy. It should be noted that the Ovito program^40^ generally does not recognize outer-layer atoms (i.e., surface atoms) as belonging to a specific local structure.

Closer examination of this nanoparticle reveals a hierarchical structure resembling a copper-coated labyrinth. The crystal lattice structure exhibits significant variability depending on the cooling rate. Analysis of Figure 5 shows that, with cooling at a rate of 0.25 K/ps, the *fcc* structure, separated by parallel hcp planes, predominates. Increasing the cooling rate to 0.5 K/ps results in the formation of individual hcp regions with cross-planes. At 0.75 K/ps, the hcp structure becomes dominant, with *fcc* planes appearing. The *bcc* lattice is less common, accounting for no more than 5% of the total volume.

For a more detailed examination of the structure, Figure 6 shows elemental maps for a Ni–Cu–Fe-Co nanoparticle consisting of 10,000 atoms at a rate of 0.25 K/ps at a final temperature of 300 K. The elemental maps provide detailed information on the spatial distribution of each component within the nanoparticle. These maps clearly show that copper exhibits pronounced surface segregation, forming a distinct outer shell around the particle. Cobalt and iron are primarily localized in the subsurface region, directly beneath the copper-enriched surface layer, indicating their tendency to occupy near-surface positions. In contrast, nickel atoms are predominantly concentrated in the central core of the nanoparticle, suggesting a compositionally labyrinth-like architecture.

**Figure 6:**
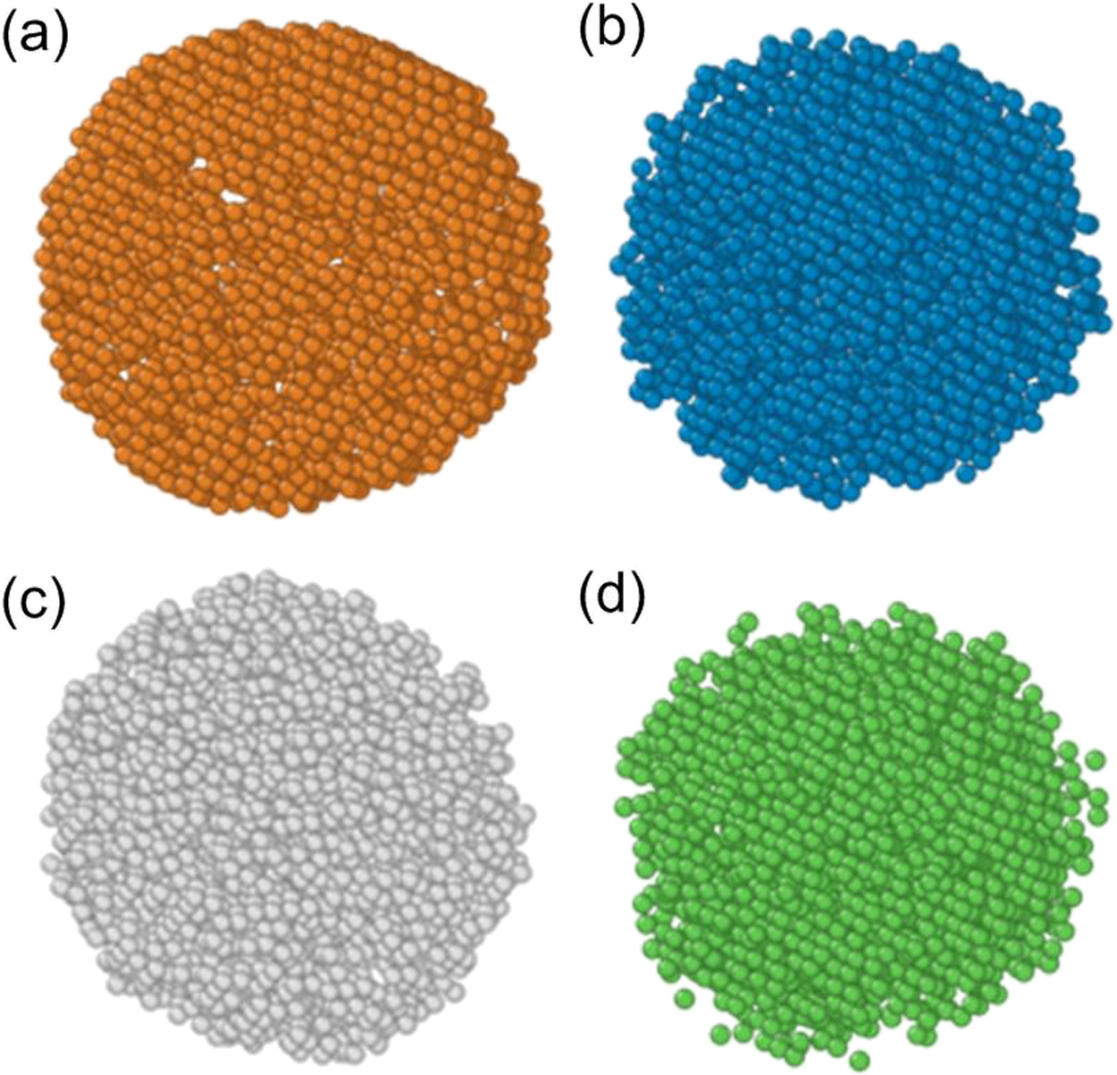
Elemental maps for the Ni–Cu–Fe–Co nanosystem consisting of 10,000 atoms for a rate of 0.25 K/ps, where (a) is copper atoms, (b) is iron, (c) is cobalt, and (d) is nickel.

For further verification of the obtained results, Figure 7 presents the temperature dependence of the potential part of the internal energy for a Ni–Cu–Fe-Co nanosystem containing 2,000 atoms, obtained using three alternative programs using different atomistic modeling methods – MD and MC.

**Figure 7:**
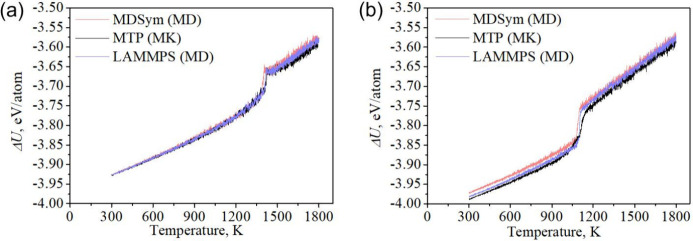
Dependence of the potential part of the specific internal energy on temperature for the Ni–Cu–Fe–Co nanosystem containing 2000 atoms at a temperature change rate of 0.5 K/ps for MD, 10^6^ MC steps, and a temperature step of 0.5 K for MC. (a) Melting; (b) cooling.

The distribution of atoms in multicomponent metallic nanoparticles, and the resulting segregation patterns, are governed by the following dominant factors:^61^

1. Relative strengths of A–A, B–B, and A–B bonds. If A–B bonds are strongest, mixing is favored; otherwise, segregation occurs, with atoms forming the strongest homonuclear bonds tending to occupy the core.
2. Surface energies of bulk elements A and B. Elements with lower surface energies tend to segregate to the surface.
3. Relative atomic sizes. Smaller atoms preferentially occupy the sterically confined core, particularly in icosahedral clusters, where core compression occurs.

The first and third factors are size-independent, whereas the second depends on nanoparticle size.^62,41^ Moreover, the rate at which surface energy approaches the macroscopic value differs for each component.

Considering all these factors in a binary system is nontrivial; in multicomponent nanoparticles, such as Ni–Cu–Fe-Co, the situation is even more complex. The atomic radii of Fe and Cu are larger than those of Ni and Co (sources differ, reporting either equal radii or a slightly larger Co radius^63,64^ ). Consequently, the behavior of a given atom type - for example, cobalt - is influenced by its relatively higher surface energy (see Table 3) and, independently, by its smaller atomic radius compared with Fe and Cu.

As can be seen from the graphs, the MC and MD methods are in good agreement. The difference in crystallization onset temperatures is within 40 K, which is less than 3.5%. Melting points for MC and MD are largely consistent. The difference in melting points does not exceed 1.5%.

### 3.2 Materials Characterization

X-ray diffraction analysis of the synthesized Ni–Cu–Fe-Co nanoparticles confirmed their high degree of crystallinity and correspondence with the simulated data. The diffraction pattern (Figure 8a) shows diffraction peaks characteristic of a face-centered cubic (*fcc*) lattice, indicating the formation of a substitutional solid solution of all four elements. The dominance of *fcc* reflections is consistent with molecular dynamics simulations, where this structure stabilized at low cooling rates. The proportion of the body-centered cubic (*bcc*) lattice, according to simulations, does not exceed 5%, and the experimental data also do not show distinct peaks.

**Figure 8:**
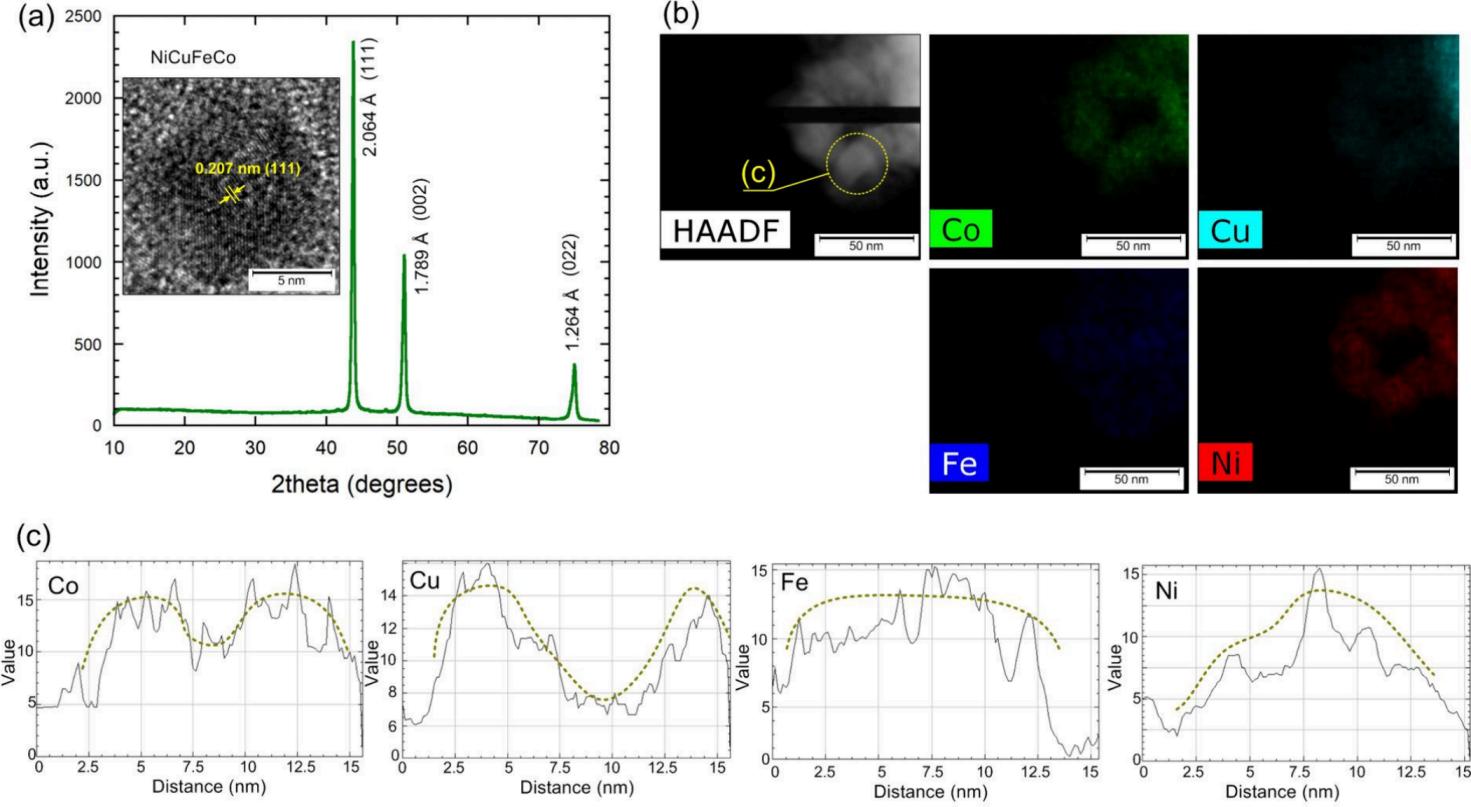
XRD pattern (a), TEM-HAADF-EDS (b), and color spectra of Co, Cu, Ni, and Fe for selected NP based on HR-TEM-EDS (obtained in ImageJ), and EDS line profiles of Co, Cu, Ni, and Fe across a representative nanoparticle (c). The green dotted line represents the averaged trend line showing the variation of elemental concentration across the nanoparticle diameter.

Morphological analysis using transmission electron microscopy (Figure 8b) revealed that the particles are spherical or slightly polyhedral, with sizes in the range of 10–30 nm, consistent with those obtained from calculations performed on systems containing 2,000–10,000 atoms. The average crystallite size, estimated from the (111) reflection using the Scherrer equation, was 24.7 nm, which agrees well with the TEM-observed particle dimensions. TEM images reveal a fairly uniform particle size distribution and the formation of aggregates typical of solution combustion synthesis products. High-resolution TEM revealed distinct crystalline domains, confirming the preservation of an ordered structure after rapid synthesis. Figures 8a and 8b demonstrate that the actual nanoparticle structure reflects the same balance of competing phases as the model results. Namely, the dominance of the fcc phase with minor hcp domains, consistent with the simulated coexistence of fcc and hcp regions at comparable cooling rates.

Elemental analysis performed using HAADF-EDS (Figure 8b and 8c) reveals maps of the distribution of chemical elements across the particle diameter. The elemental spectra (Figure 8c) clearly show the uneven spatial distribution: copper is predominantly concentrated on the surface of the nanoparticles, while nickel and iron form the inner core. The apparent increase of Cu intensity near 2.5–5 nm in the EDS line profile is attributed to projection overlap of Cu-enriched surface regions in the TEM cross-section. This feature agrees with the simulated radial Cu density maximum near the outer shell, confirming surface-driven Cu segregation. Cobalt exhibits intermediate behavior, partially enriching the periphery but retaining a significant fraction in the bulk. This distribution also agrees well with the results of molecular dynamics and Monte Carlo simulations, which observed stable segregation of copper into the shell and the formation of a core of Ni and Fe.

The formation of a copper shell reduces the surface energy of the nanoparticles, which is consistent with both theoretical calculations and literature data on the tendency of Cu to undergo surface segregation due to low cohesive energy. Similarly, the observed distribution of Co reflects its intermediate nature: some cobalt atoms migrate to the surface, stabilizing the shell, while others are anchored in the core along with Ni and Fe, thereby enhancing structural stability. This result experimentally confirms the conclusion made in Section 3.1 about three types of atoms in a multicomponent system: with a pronounced tendency toward segregation (Cu), with a preference for the bulk phase (Ni, Fe), and those with intermediate behavior (Co).

## 4 Conclusions

Molecular dynamics simulations allowed us to characterize in detail the behavior of equiatomic Ni–Cu–Fe-Co nanoparticles during phase transitions. It was found that the melting onset and crystallization completion temperatures (*T*_m_ and *T*_c_, respectively) depend on both the system size and the rate of temperature change. Increasing nanoparticle size leads to an increase in melting temperature and a decrease in surface energy, indicating increased structural stability with increasing particle size. Increasing the cooling rate significantly decreases the crystallization temperature and increases the difference between *T*_m_ and *T*_c_, indicating a significant influence of kinetic factors on the crystallization mechanism.

Size effects are observed not only in changes of thermodynamic properties but also in energy parameters: as the size of the particle increases, the potential energy becomes more negative, consistent with increased volume fraction of stable crystalline domains. With the increase in size, there is a corresponding decrease in surface energy resulting in smaller contributions from defects and disordered regions. However, the choice of dividing surface significantly affects the surface energy of a nanoparticle, therefore, the data in this study are estimates. Chemical and structural segregation exhibit a robust dependence on both particle size and cooling rate. Regardless of these parameters, a copper- and cobalt-enriched shell forms on the surface, while the core is predominantly composed of iron and nickel. The structural composition also changes: the proportion of different crystalline phases (*fcc*, *hcp*, *bcc*) varies with cooling rate, reflecting structure-dependent rearrangements, especially between *fcc* and hcp phases. During slow cooling, the *fcc* structure predominates, while at high cooling rates, *hcp* regions form preferentially. Furthermore, it has been shown that, according to the classification,^23–25^ Cu and Co atoms exhibit a tendency toward surface segregation, Ni and Fe atoms form the core, and Co atoms are partially distributed between the core and the shell.

The results demonstrate strong agreement between the experimental and calculated results. XRD confirmed the predominance of an *fcc* structure with *hcp* fragments, TEM revealed a nanocrystalline character, and TEM-EDS confirmed pronounced segregation of copper and cobalt on the surface. All these observations are fully consistent with simulations, where similar patterns were observed depending on nanoparticle size and cooling rate.

## Supplementary Material



## Data Availability

Data will be made available on request.

## Notes

Additional low-magnification TEM images of the synthesized Ni–Cu–Fe–Co nanoparticles (PDF)
