# Supplementary material for: Chemical and Structural Segregation in Quaternary Ni–Cu–Fe-Co Nanoparticles: Atomistic Simulation and Experiment
Source: ACS Phys Chem Au. 2025 Dec 8;6(1):163–73. doi: 10.1021/acsphyschemau.5c00102 (PMC12856664; doi:10.1021/acsphyschemau.5c00102)
Supplement: Supplementary file 1 [file pg5c00102_si_001.pdf]

Chemical and Structural Segregation in Quaternary Ni-Cu-Fe-Co Nanoparticles: Atomistic Simulation and Experiment

Andrey Yu. Kolosov [1], Nikita Nepsha [1], Denis Sokolov [1], Kseniya G. Savina [1], Dmitry Moskovskikh [2], Evgenii Beletskii [3], Saravana Kumar M. [4,5], Nickolay Yu. Sdobnyakov [1,*], Valentin Romanovski [6,*]

[1] Department of General Physics, Tver State University, Tver, Russia

[2] Science and Research Centre of Functional Nano-Ceramics, National University of Science and Technology "MISIS", Moscow, Russia

[3] MllT Key Laboratory of Critical Materials Technology for New Energy Conversion and Storage, School of Chemistry and Chemical Engineering, Harbin Institute of Technology, Harbin 150001, China

[4] Graduate Institute of Manufacturing Technology, National Taipei University of Technology, Taipei 10608, Taiwan.

[5] Department of Mechanical Engineering, Saveetha School of Engineering, Saveetha Institute of Medical and Technical Sciences (SIMATS), Chennai, Tamil Nadu 602105, India.

[6] Department of Materials Science and Engineering, University of Virginia, Charlottesville, USA.

Additional TEM images in better resolution

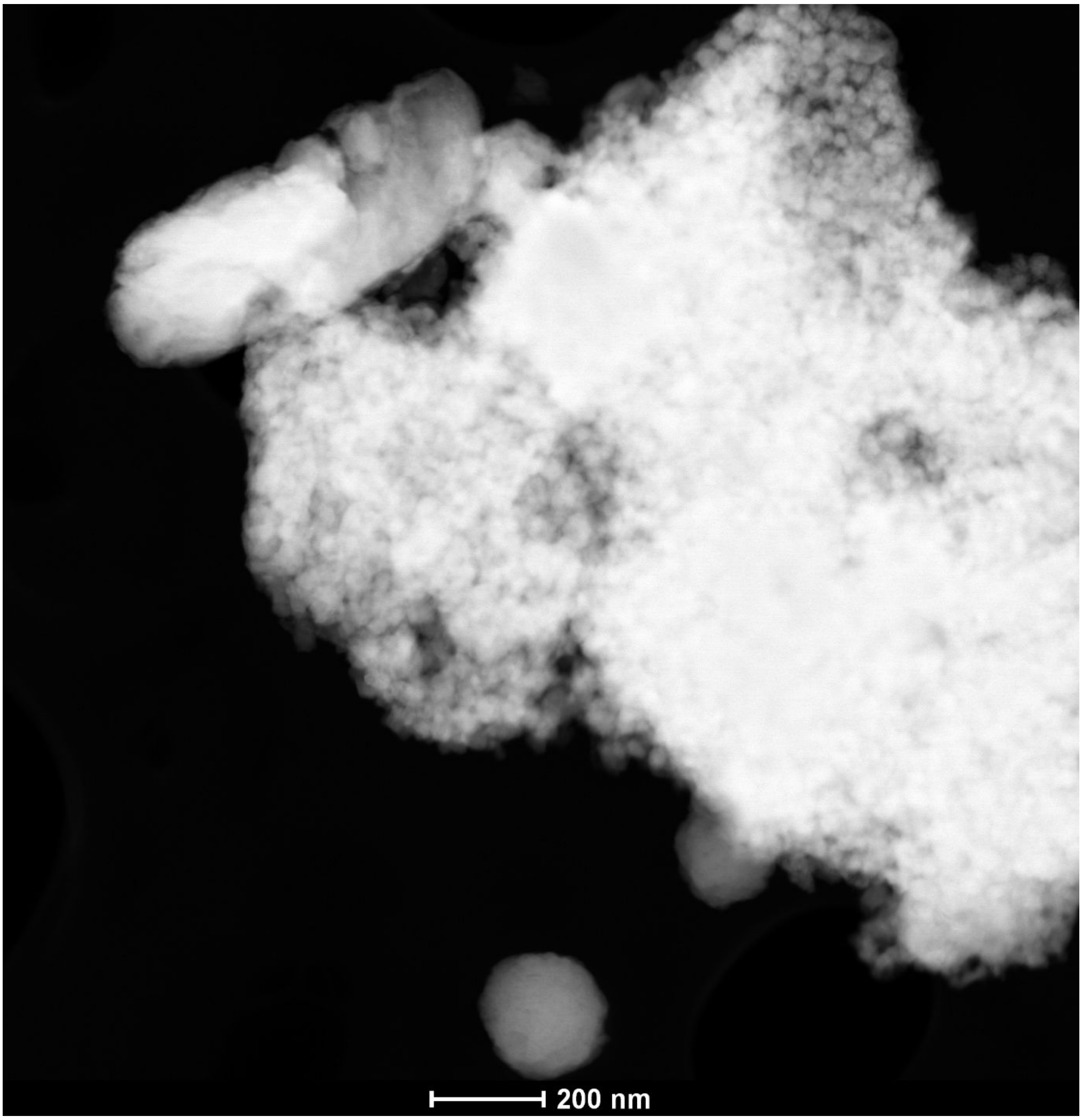
200 nm

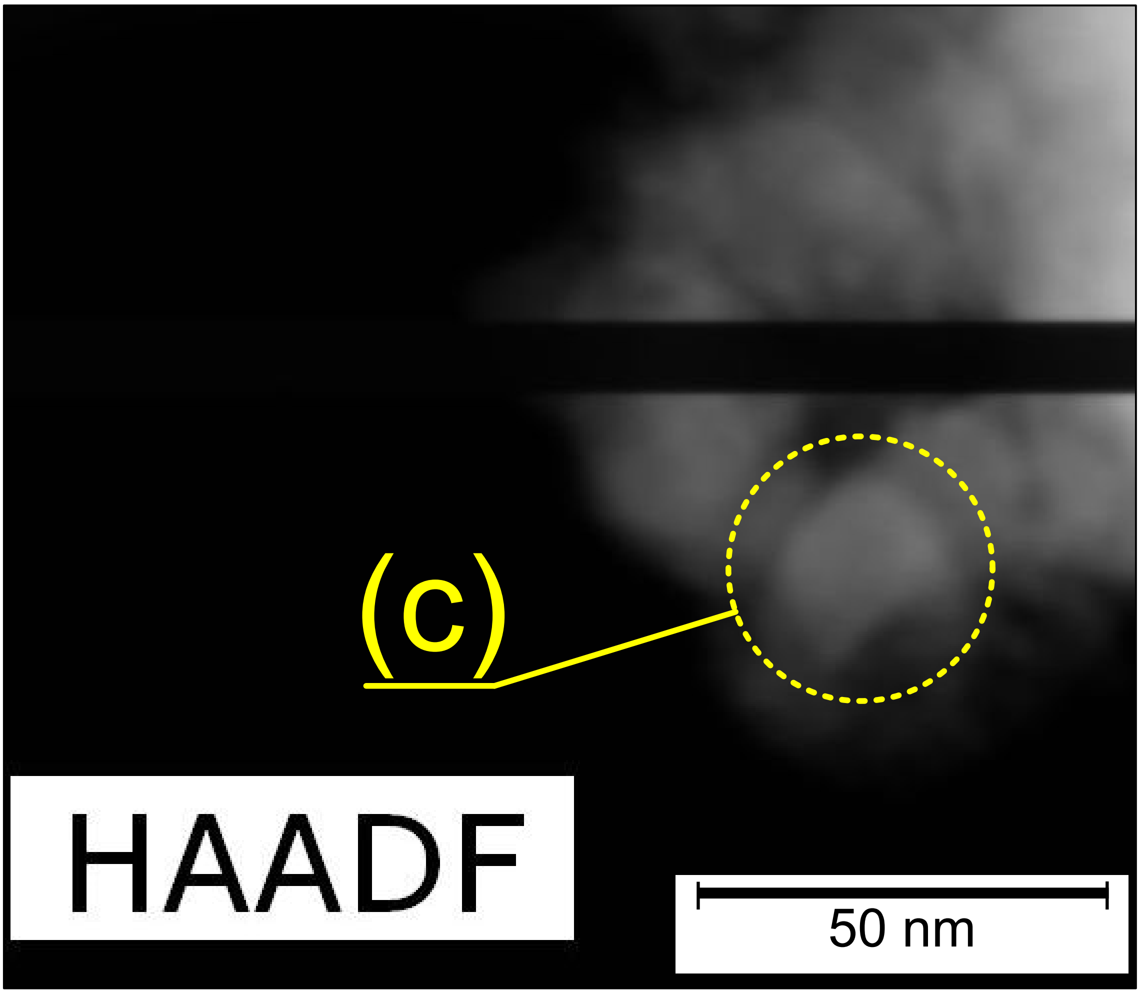
(c)
HAADF
50 nm

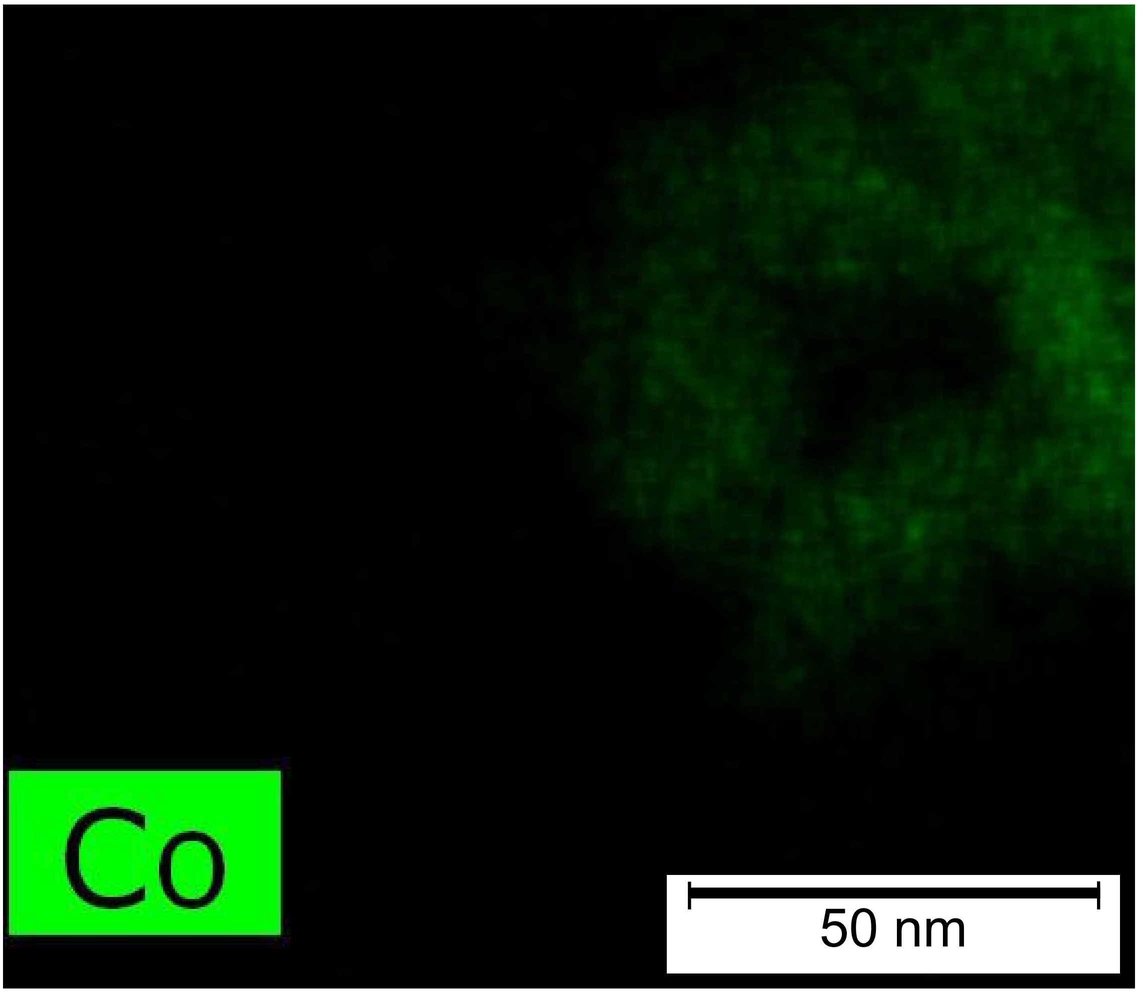
Co
50 nm

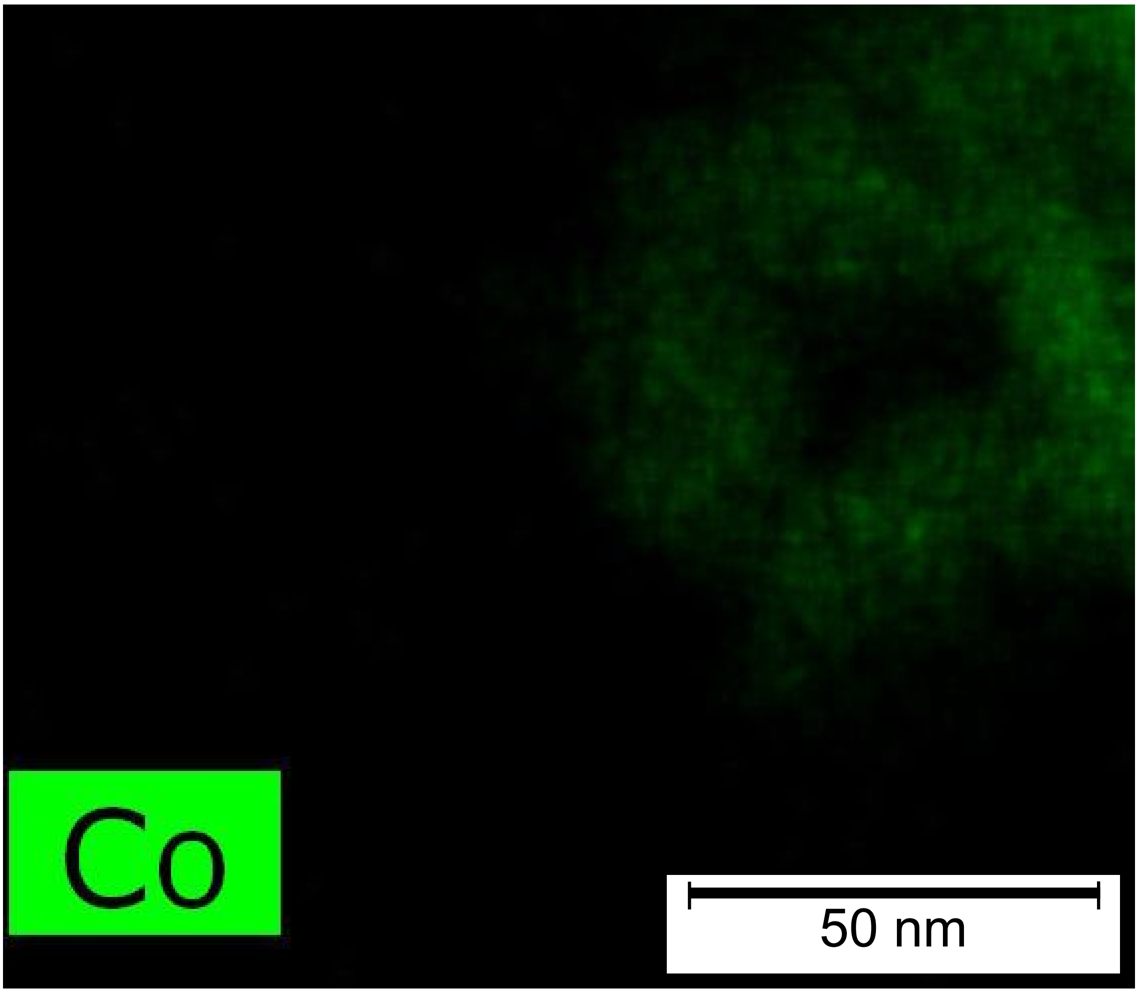
Co
50 nm

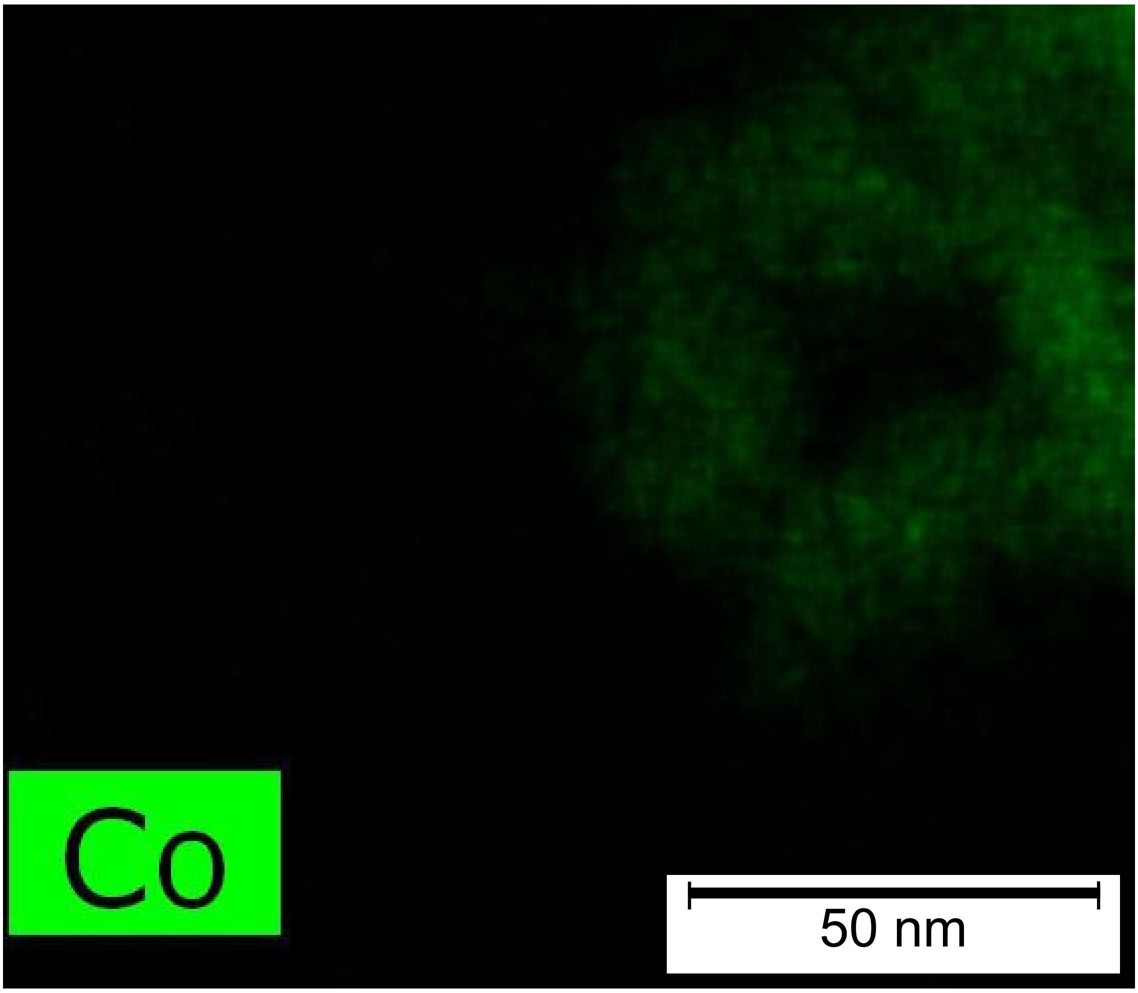
Co
50 nm

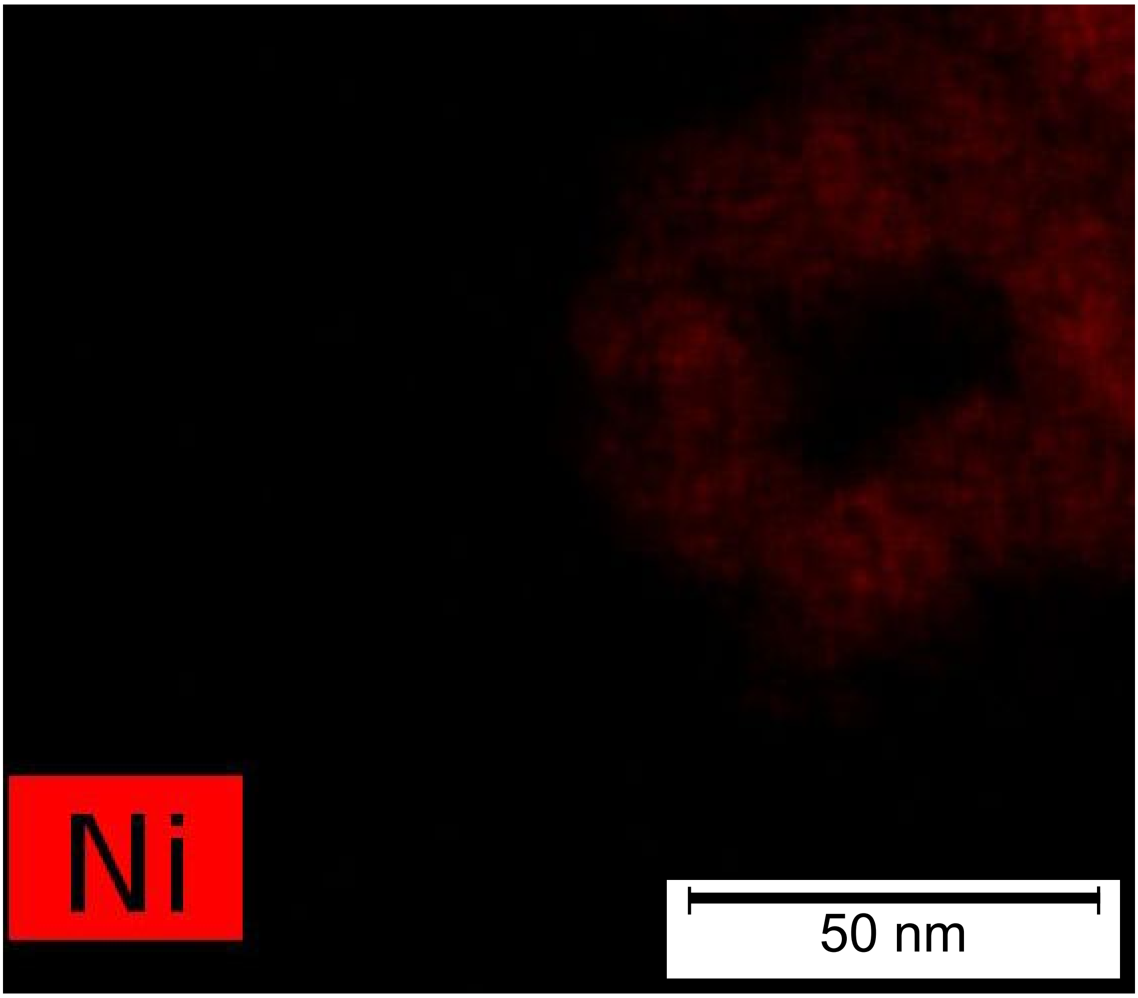

Figure S1. Low-magnification TEM images of the synthesized Ni-Cu-Fe-Co nanoparticles
